# Editorial: Advances in myeloid cell targeting for overcoming tumor immunosuppression

**DOI:** 10.3389/fimmu.2025.1737187

**Published:** 2025-11-13

**Authors:** Dehong Yan, Qiong Yang, Jingying Zhou

**Affiliations:** 1 Guangdong Immune Cell Therapy Engineering and Technology Research Center, Center for Protein and Cell-Based Drugs, Institute of Biomedicine and Biotechnology, Shenzhen Institutes of Advanced Technology, Chinese Academy of Sciences, Shenzhen, China; 2 School of Medicine, South China University of Technology, Guangzhou, China; 3 School of Biomedical Sciences, The Chinese University of Hong Kong, Hong Kong, Hong Kong SAR, China

**Keywords:** myeloid cells, tumor immunosuppression, immunotherapy resistance, cancer treatment, myeloid-derived suppressor cells (MDSCs)

This Research Topic, *Advances in myeloid cell targeting for overcoming tumor immunosuppression*, comprises one comprehensive review and three original research articles, which collectively explore recent advances in myeloid cell targeting approaches, including nanoparticle-based siRNA and engineered oncolytic viruses. Mechanistically, these new approaches lead to the reprogramming of myeloid-derived suppressor cells (MDSCs) and tumor-associated macrophages (TAMs), thereby enhancing anti-tumor immunity. Moreover, imaging-based biomarkers like imaging-based myeloid response score (iMRS) show promise for patient stratification. Despite progress, challenges remain in overcoming myeloid plasticity, toxicity, and developing reliable biomarkers for effective combination therapies.

Lang et al. developed peptide-based nanoparticles (NPc-Rel) to deliver siRNA targeting the transcription factor c-Rel, a myeloid immune checkpoint, in MDSCs. NPc-Rel effectively silenced c-Rel expression, reduced MDSC numbers and immunosuppressive function, and enhanced CD8^+^ T cell activity in the tumor microenvironment. In a B16 melanoma mouse model, this strategy significantly inhibited tumor growth, demonstrating that targeting myeloid c-Rel with siRNA nanoparticles is a promising immunotherapy approach to overcome tumor immunosuppression.

Jennings et al. demonstrated that oncolytic rhabdoviruses (ORVs) armed with microRNAs can reprogram TAMs via tumor-derived extracellular vesicles (TDEVs). ORV infection enhanced TDEV production, which was preferentially taken up by myeloid cells. ORVs expressing miR-155 or miR-19a delivered these miRNAs to TAMs, reversing their immunosuppressive function and enhancing T cell activity. While ORV-miR-155 improved antigen presentation and T cell responses, ORV-miR-19a significantly prolonged survival in a murine ovarian cancer model. This approach effectively reprograms the immunosuppressive tumor microenvironment, enhancing the efficacy of oncolytic virotherapy.

Peng et al. developed a noninvasive iMRS using preoperative CT radiomics to predict the myeloid immune contexture in hepatocellular carcinoma (HCC). A multi-phase CT radiomics signature accurately predicted the pathological MRS, which reflects the balance of pro- and anti-tumor myeloid cells. A high iMRS was significantly associated with poor post-surgical overall and recurrence-free survival. Furthermore, in an immunotherapy cohort, a high iMRS predicted a better objective response and improved survival for patients treated with anti-PD-1/PD-L1 therapy, demonstrating its value as a noninvasive biomarker for prognosis and immunotherapy benefit assessment.

The review by Chen et al. summarizes the potential of targeting myeloid cells to overcome immunosuppression in the tumor microenvironment (TME) and enhance cancer immunotherapy. Myeloid populations—such as TAMs, MDSCs, and tumor-associated dendritic cells—promote immune evasion through cytokine secretion, metabolic reprogramming, immune checkpoint expression, and physical exclusion of T cells. The article highlights key therapeutic targets, including CSF1R, PI3Kγ, mTOR, Syk, MerTK/Axl, and immune checkpoints like TREM2, LILRBs, VISTA, and CD40. Preclinical and clinical evidence supports strategies to deplete or reprogram these cells, restoring anti-tumor immunity. Combining myeloid-targeted agents with checkpoint inhibitors, chemotherapy, or radiation shows synergistic promise. However, challenges such as myeloid plasticity, toxicity, and biomarker development remain. Future success hinges on personalized approaches and rational combination therapies.

Future research directions in targeting myeloid cells to overcome tumor immunosuppression will focus on several key areas. A major priority is the development of more selective agents and delivery systems, such as nanoparticles or bispecific antibodies, to precisely modulate specific myeloid subpopulations like TAMs or MDSCs without systemic toxicity. Understanding and targeting the metabolic and epigenetic reprogramming that sustains the immunosuppressive functions of these cells represent another critical avenue. Furthermore, identifying robust predictive biomarkers—through single-cell sequencing or spatial transcriptomics—is essential for patient stratification and for monitoring therapy-induced changes in the myeloid compartment in real-time. Finally, a central challenge is the rational design of combination therapies. This involves integrating myeloid-targeting agents with existing immunotherapies (e.g., checkpoint inhibitors, CAR-T), conventional treatments, or novel modalities like STING agonists to achieve synergistic efficacy, reverse resistance, and create a durable anti-tumor immune response in a wider range of cancers.

